# Psychobehavioral Assessment and Brief Cognitive–Behavioral Therapy in Resistant Arterial Hypertension: A Feasibility-Oriented Pilot Study Within a Precision Medicine Framework

**DOI:** 10.3390/jpm16060293

**Published:** 2026-05-28

**Authors:** Apoenna Marina Noronha Brito, Enilson Carmo Barbosa Dos Santos, Andre Rodrigues Duraes, Carla Daltro

**Affiliations:** University Hospital Complex Prof. Edgard Santos, Federal University of Bahia, Rua Augusto Viana S/N, Salvador 40110-060, Brazil; enilson.santos@ebserh.gov.br (E.C.B.D.S.); andre.duraes@ebserh.gov.br (A.R.D.); carla.daltro@ebserhnet.onmicrosoft.com (C.D.)

**Keywords:** resistant hypertension, cognitive–behavioral therapy, anxiety, depression, behavioral cardiology, precision medicine, feasibility study

## Abstract

**Background:** Resistant arterial hypertension (RAH) is a heterogeneous cardiovascular condition influenced by biological, behavioral, psychosocial, and neuroendocrine mechanisms. Within emerging precision medicine frameworks, psychobehavioral assessment may contribute to a more individualized characterization of patients with RAH and help identify modifiable dimensions associated with therapeutic resistance. This study evaluated the feasibility and preliminary outcomes of a brief psychobehavioral intervention in patients with RAH. **Methods:** This feasibility-oriented exploratory pre–post pilot study included 20 adults with RAH recruited from a tertiary outpatient clinic specialized in resistant hypertension. Participants underwent psychobehavioral assessment using the Hospital Anxiety and Depression Scale (HADS). Individuals presenting clinically significant anxiety and/or depressive symptoms (scores ≥ 8) received an individualized semi-structured brief cognitive–behavioral therapy (CBT) intervention consisting of 8–9 weekly sessions. Feasibility indicators included intervention adherence, completion of the protocol, operational flexibility, and absence of symptom worsening. Pre- and post-intervention emotional symptoms were compared using nonparametric analyses. **Results:** High baseline emotional burden was observed, with 90% of participants presenting anxiety symptoms and 60% depressive symptoms. Following the intervention, reductions in anxiety [median 11 (IQR 8–13) vs. 6 (4–8); *p* < 0.001] and depressive symptoms [10 (8–11) vs. 5 (3–8); *p* < 0.001] were identified. No worsening of symptoms occurred. The intervention demonstrated satisfactory feasibility and acceptability, including flexibility for remote and in-person delivery. **Conclusions:** These preliminary findings suggest that psychobehavioral phenotyping combined with individualized brief CBT may represent a feasible complementary strategy within precision-oriented cardiovascular care for resistant hypertension. Although causal inference cannot be established due to the pilot design and absence of a control group, the findings support further investigation of psychobehavioral dimensions as potentially relevant components of personalized hypertension management.

## 1. Introduction

Systemic arterial hypertension remains one of the most prevalent and clinically impactful cardiovascular conditions worldwide. It is characterized by a multifactorial etiology, frequently silent progression, and progressive target-organ damage involving the heart, kidneys, brain, and vasculature [1,2,3,4]. Epidemiological data from Brazil indicate a sustained increase in hypertension prevalence, reinforcing its persistent public health burden and the need for therapeutic strategies that extend beyond pharmacological intensification [4].

Resistant arterial hypertension (RAH) represents a clinically complex condition within the hypertensive spectrum. It is commonly defined as uncontrolled blood pressure despite optimized treatment with three antihypertensive drug classes, including a diuretic, or controlled blood pressure requiring four or more agents [1,5]. Rather than a uniform pharmacological failure, RAH is increasingly recognized as a heterogeneous condition integrating hemodynamic, neurohormonal, metabolic, psychosocial, and behavioral mechanisms [6,7,8]. Sympathetic overactivity is considered a central contributor, promoting sustained vasoconstriction, arterial stiffness, and persistent elevation of peripheral resistance [9].

Psychological distress has emerged as a relevant factor in cardiovascular regulation. Anxiety and depressive symptoms have been associated with resistant hypertension, increased blood pressure variability, autonomic imbalance, treatment non-adherence, and adverse cardiovascular outcomes [10,11,12,13,14]. Chronic psychosocial stress may activate both the sympathetic nervous system and the hypothalamic–pituitary–adrenal axis, contributing to endothelial dysfunction, cortisol dysregulation, sodium retention, inflammation, and increased vascular resistance [9,15,16].

Within emerging precision medicine perspectives, there is increasing recognition of the importance of understanding the individual as a whole, integrating biological, behavioral, and psychosocial dimensions in clinical decision-making. Precision medicine in cardiovascular disease increasingly recognizes that therapeutic response variability cannot be fully explained by traditional biological markers alone. Behavioral regulation, emotional distress, coping patterns, treatment adherence, and psychosocial stress exposure may contribute to clinically meaningful heterogeneity among patients with resistant hypertension. In this context, psychobehavioral phenotyping may represent a complementary strategy for identifying potentially modifiable dimensions associated with therapeutic resistance and differential clinical trajectories. Rather than replacing conventional cardiovascular management, this approach seeks to integrate psychosocial and behavioral information into a broader individualized care model [16,17,18,19]. Accordingly, precision-oriented cardiovascular care moves beyond standardized therapeutic models and emphasizes individual variability in disease expression, treatment response, and health-related behaviors [17].

Within this context, identifying potentially modifiable contributors underlying therapeutic resistance may support more individualized cardiovascular care [17]. Cognitive–behavioral therapy (CBT), grounded in the restructuring of maladaptive cognitive patterns and emotional regulation processes, has well-established efficacy for anxiety and depressive symptoms [18,19]. Evidence also suggests that CBT-based interventions may be associated with reductions in blood pressure parameters and improvements in psychological outcomes in hypertensive patients [20].

Considering the multifactorial nature of resistant arterial hypertension, standardized therapeutic approaches may be insufficient to address the complexity and interindividual variability of this condition. Increasing evidence suggests that emotional distress, stress-related neuroendocrine dysregulation, maladaptive coping, and behavioral barriers to adherence may contribute to therapeutic resistance and variability in clinical outcomes. Despite growing recognition of mind–body interactions in cardiovascular disease, structured psychobehavioral assessment remains underexplored in resistant hypertension. Within emerging precision-oriented cardiovascular care models, these dimensions may represent relevant complementary targets for individualized therapeutic strategies.

Therefore, this study aimed to evaluate the feasibility, acceptability, and preliminary outcomes of a brief CBT intervention informed by psychobehavioral assessment in patients with resistant arterial hypertension.

## 2. Materials and Methods

### 2.1. Study Design and Participants

This was a feasibility-oriented, exploratory pre–post pilot intervention study conducted between March 2023 and May 2024 at a university hospital outpatient clinic specialized in resistant hypertension. Twenty adults diagnosed with RAH were included. All participants provided informed consent.

As a pilot study, no formal sample size calculation was performed. The sample was considered adequate to explore feasibility and preliminary signals of change.

### 2.2. Psychobehavioral Assessment

Participants were screened using the Hospital Anxiety and Depression Scale (HADS). Individuals scoring ≥ 8 in anxiety and/or depression were considered to present clinically relevant affective symptoms and were invited to participate in the psychotherapeutic phase [21].

### 2.3. Outcomes

Primary outcomes included feasibility and acceptability of the intervention protocol. Feasibility indicators included successful delivery of the planned sessions, completion of the psychotherapeutic phase, and absence of symptom worsening. Acceptability was explored through participant adherence, engagement throughout the intervention, and the operational flexibility of session delivery, including in-person and remote modalities according to participant availability. Secondary outcomes included changes in anxiety and depressive symptoms assessed by the HADS.

### 2.4. Intervention

**Figure 1:**
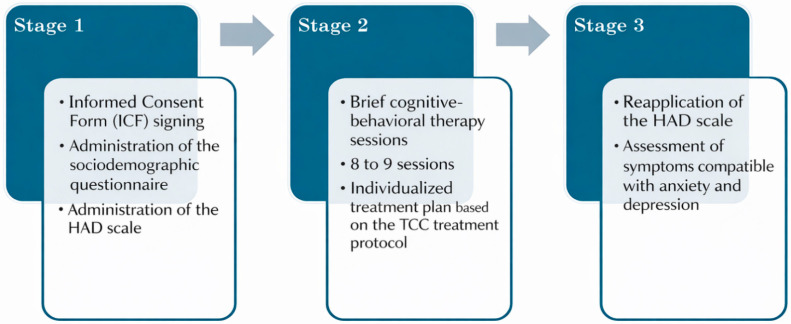
Flowchart of the three-stage cognitive–behavioral intervention protocol and assessment process. Salvador, Bahia, Brazil, 2024.

The intervention was delivered by a trained clinical psychologist with experience in cognitive–behavioral therapy (CBT) and cardiovascular-related psychological care. Participants underwent an individualized brief CBT protocol consisting of 8–9 weekly sessions lasting approximately 50 min each, conducted either in person or remotely according to participant availability and logistical feasibility (Figure 1).

Sessions followed a semi-structured format based on established CBT principles, although the intervention was not derived from a fully manualized protocol [22]. To improve reproducibility, the intervention followed a structured therapeutic sequence while maintaining flexibility according to individual psychobehavioral characteristics and emotional demands identified during clinical assessment.

Session 1 focused on clinical interview, psychobehavioral assessment, collaborative treatment planning, establishment of the therapeutic confidentiality agreement, and identification of the main emotional and behavioral demands associated with hypertension management. An individualized problem list was developed to guide psychotherapy and support psychobehavioral formulation. These procedures aimed to facilitate the interruption of maladaptive maintenance cycles associated with anxiety and depressive symptoms.

Sessions 2–3 emphasized psychoeducation regarding the interaction between emotional stress, cognition, behavioral responses, and cardiovascular symptoms. During these sessions, dysfunctional automatic thoughts, maladaptive beliefs, emotional triggers, and behavioral patterns associated with psychological distress were identified and monitored.

Sessions 4–6 focused on cognitive restructuring, behavioral activation, emotional regulation, and coping-skills training. Intervention components were selected according to individual patient needs and included pleasant activity scheduling, thought records, identification and restructuring of dysfunctional automatic thoughts, decisional balance exercises addressing advantages and disadvantages of maintaining maladaptive beliefs, coping cards, downward arrow technique, cognitive continuum exercises, belief modification strategies, stress management techniques, and problem-solving training.

Sessions 7–8 emphasized consolidation of adaptive coping strategies, reinforcement of therapeutic gains, relapse prevention, emotional self-monitoring, and identification of daily triggers associated with anxiety and depressive symptoms. Participants were also encouraged to adopt behavioral and lifestyle strategies potentially associated with improved emotional well-being, quality of life, emotional regulation, and treatment adherence.

When a ninth session was required, it was used for individualized reinforcement, therapeutic closure, and final review of coping strategies and relapse-prevention techniques.

Following completion of the intervention, the Hospital Anxiety and Depression Scale (HADS) was reapplied to assess post-intervention emotional symptoms and compare pre- and post-intervention scores.

### 2.5. Statistical Analysis

Statistical analyses were descriptive and exploratory. Pre–post comparisons were performed using the Wilcoxon signed-rank test. Results are presented as medians and interquartile ranges.

Given the small sample size, results should be interpreted cautiously and are not intended to support inferential generalization.

Effect sizes were estimated to provide an indication of the magnitude of change. Confidence intervals were not calculated due to the exploratory pilot design and small sample size.

## 3. Results

Feasibility was supported by the successful delivery of the 8–9 session protocol, absence of symptom worsening, and completion of the intervention among participants who entered the psychotherapeutic phase. To facilitate adherence, sessions were conducted either in person or remotely according to participant availability and logistical needs. Some individuals initially screened for participation were not included in the psychotherapeutic phase due to outdated contact information or failure to attend the initial psychological intake interview.

Twenty participants were included (Table 1). The median age was 55 years (IQR: 48–64). Most participants were female, had a partner, had children, and all self-identified as Afro-descendant. Regarding socioeconomic characteristics, most participants reported a family income between one and two minimum wages and had completed secondary education (Table 1).

At baseline, clinically significant emotional distress was highly prevalent. Eighteen participants (90%) presented symptoms compatible with anxiety, 12 (60%) presented depressive symptoms, and 10 (50%) exhibited concomitant anxiety and depression. Considerable interindividual variability in emotional symptom burden was observed across participants, reinforcing the heterogeneous psychobehavioral profile of patients with resistant hypertension. These findings support the relevance of incorporating psychobehavioral dimensions into broader individualized cardiovascular assessment models [17,18].

Following the psychotherapeutic intervention, reductions in symptom scores were observed in both anxiety and depressive symptoms. Among the 18 participants presenting anxiety symptoms at baseline, 13 (72.2%) showed reduced symptoms, while five (27.8%) showed no change. Among the 12 participants with depressive symptoms, seven (58.3%) improved and five (41.7%) remained stable. No participants exhibited worsening of symptoms, and no new cases of anxiety or depression were identified after the intervention.

**Figure 2:**
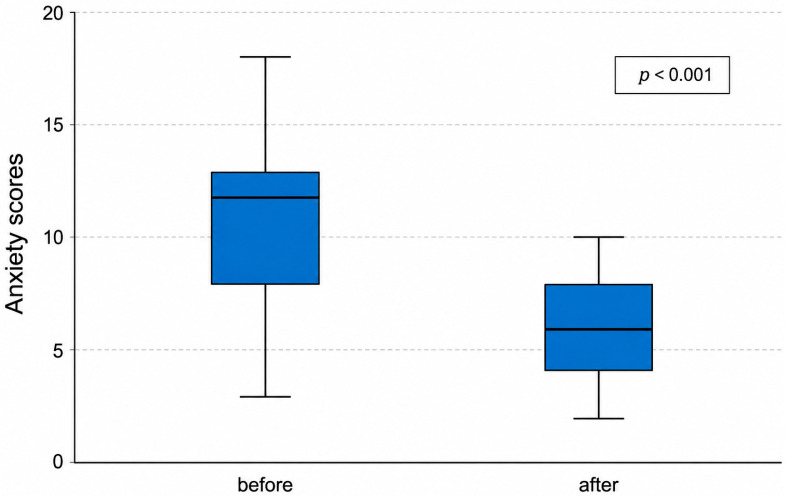
Box plot illustrating the distribution of HADS anxiety scores before and after the intervention. Median values, interquartile ranges, and variability are presented. A statistically significant reduction in anxiety symptoms was observed following the intervention (*p* < 0.001). Salvador, Bahia, Brazil, 2024.

**Figure 3:**
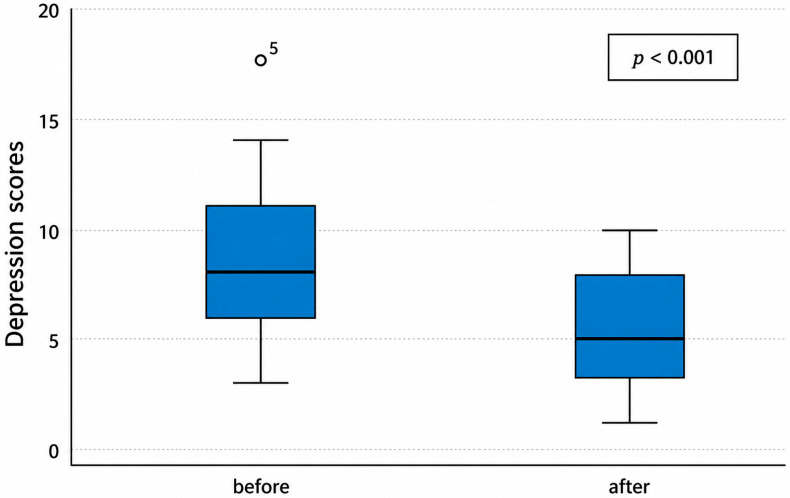
Box plot illustrating the distribution of HADS depressive symptom scores before and after the intervention. Median values, interquartile ranges, and variability are presented. A statistically significant reduction in depressive symptoms was observed following the intervention (*p* < 0.001). Salvador, Bahia, Brazil, 2024.

Consistent with these findings, significant reductions in HADS scores were observed after the intervention. Median anxiety scores decreased from 11 (IQR: 8–13) at baseline to 6 (IQR: 4–8) post-intervention (*p* < 0.001). Depressive symptom scores also decreased significantly, from 10 (IQR: 8–11) to 5 (IQR: 3–8) (*p* < 0.001) (Figure 2 and Figure 3).

## 4. Discussion

This pilot study demonstrates a high prevalence of anxiety and depressive symptoms among patients with RAH and suggests that a brief individualized cognitive–behavioral therapy (CBT) protocol may represent a feasible approach within cardiovascular care. However, given the exploratory design, these findings should be interpreted cautiously and do not allow causal inference.

The present findings also support the growing recognition that psychobehavioral characteristics may contribute to variability in clinical presentation in RAH, highlighting their potential relevance in the broader characterization of this condition.

RAH is increasingly conceptualized as a heterogeneous clinical condition influenced by complex interactions between biological, behavioral, and environmental determinants [1,2,3]. Within contemporary precision medicine frameworks, resistant hypertension is increasingly understood as a multidimensional condition involving biological, behavioral, psychosocial, and environmental interactions [16,17,18,19]. Although current precision cardiovascular approaches traditionally emphasize molecular and genomic markers, there is growing recognition that psychobehavioral characteristics may also influence disease trajectories, treatment adherence, autonomic regulation, and therapeutic responsiveness. In this context, psychobehavioral assessment may contribute to a broader individualized characterization of patients with resistant hypertension, particularly when emotional distress and maladaptive coping patterns are clinically relevant.

The present findings support the hypothesis that anxiety and depressive symptoms may represent potentially actionable dimensions within resistant hypertension management. While no formal phenotypic stratification was performed, the high prevalence of emotional symptoms observed in this cohort reinforces the importance of integrating psychosocial dimensions into individualized cardiovascular care models.

In the present study, the demographic and clinical profile of the participants reflected characteristics previously associated with resistant hypertension. Obesity and Afro-descendant ethnicity were characteristic features within the sample. Previous evidence suggests that Afro-descendant populations may present a higher prevalence of resistant hypertension and differential responses to antihypertensive therapies, potentially reflecting complex genetic, environmental, and socioeconomic determinants [23]. In addition, obesity represents an important modifiable risk factor, as weight reduction has been associated with reductions in blood pressure levels and a lower likelihood of developing resistant hypertension [14]. These findings reinforce the multifactorial nature of RAH and highlight the importance of considering clinical and demographic heterogeneity when interpreting treatment responses.

Beyond these clinical determinants, our findings emphasize the relevance of psychobehavioral factors in this population. The high baseline prevalence of anxiety (90%) and depressive symptoms (60%) observed in this cohort suggests that emotional distress may represent a frequent and relevant dimension in patients with resistant hypertension. These results are consistent with previous studies reporting associations between anxiety and depressive disorders and resistant hypertension [4].

Following the psychotherapeutic intervention, reductions in emotional symptom scores were observed. These changes should be interpreted with caution, as the absence of a control group prevents the exclusion of alternative explanations. The observed changes may also be influenced by non-specific factors such as therapeutic interaction, expectancy effects, and regression to the mean.

Previous studies have demonstrated that CBT-based approaches may be associated with reductions in psychological distress and improvements in health-related behaviors in patients with chronic cardiovascular conditions. Recent evidence from a systematic review and meta-analysis by Wang et al. demonstrated that internet-based CBT interventions were associated with significant reductions in anxiety and depressive symptoms among patients with cardiovascular and cerebrovascular diseases, reinforcing the potential relevance of psychobehavioral interventions in cardiovascular populations [24].

From a mechanistic perspective, sympathetic nervous system overactivity has been described as an important pathway in the pathophysiology of hypertension [7]. Chronic anxiety and psychological stress may be associated with sustained autonomic activation and dysregulation of the hypothalamic–pituitary–adrenal axis, contributing to endothelial dysfunction and increased vascular resistance [6,7]. Emerging evidence also suggests that psychobehavioral interventions may influence stress-related biological pathways, including inflammatory signaling, neuroendocrine regulation, and epigenetic mechanisms such as DNA methylation, reinforcing the potential biological interface between emotional regulation and cardiovascular health [25]. However, no physiological or biomarker data were collected in the present study, and mechanistic interpretations remain speculative.

Although some studies suggest that CBT-based interventions may be associated with reductions in blood pressure parameters [10], such effects were not directly evaluated in the present study and should not be inferred.

Within emerging precision medicine perspectives, psychobehavioral assessment may represent a complementary dimension for characterizing patients with resistant hypertension. However, no formal stratification or phenotype-driven approaches were applied in this study.

These findings are also consistent with emerging evidence supporting digitally delivered psychological interventions as complementary strategies in cardiovascular care, particularly for addressing emotional distress and behavioral regulation in chronic cardiovascular conditions [26]. A recent systematic review and network meta-analysis by Ding et al. demonstrated that digital psychological interventions may contribute to improvements in psychological outcomes among cardiovascular patients, reinforcing the potential relevance of flexible and accessible psychobehavioral care models within individualized cardiovascular management frameworks [26].

The absence of symptom worsening supports the tolerability of the intervention. However, statements regarding scalability or broad clinical applicability should be interpreted cautiously given the pilot nature of the study.

From a clinical perspective, the operational flexibility and tolerability observed in this pilot study may support the future incorporation of structured psychobehavioral assessment into multidisciplinary resistant hypertension care programs. Although preliminary, these findings suggest that individualized psychological interventions may help address emotional distress and behavioral barriers potentially associated with therapeutic resistance. Future precision-oriented cardiovascular models may benefit from integrating psychobehavioral dimensions alongside conventional clinical and biological assessments.

### Limitations

This study has several important limitations. The small sample size and absence of a control group limit causal inference. The inclusion of participants with elevated HADS scores may introduce selection bias and regression to the mean. No follow-up assessments were conducted, preventing evaluation of sustained effects. Additionally, no objective clinical outcomes, such as blood pressure measurements, were included.

Future studies should include controlled designs, longitudinal follow-up, and objective physiological measures to better understand the clinical relevance of psychobehavioral interventions in resistant hypertension.

Taken together, these findings provide preliminary evidence that psychobehavioral factors may represent a relevant dimension for future investigation in resistant arterial hypertension, although their clinical implications remain to be determined.

## 5. Conclusions

This feasibility-oriented pilot study provides preliminary evidence that psychobehavioral assessment combined with individualized brief cognitive–behavioral therapy is feasible and well tolerated among patients with resistant arterial hypertension. Significant reductions in anxiety and depressive symptoms were observed following the intervention, suggesting that emotional distress may represent a clinically relevant dimension within the heterogeneous phenotype of resistant hypertension.

Within emerging precision-oriented cardiovascular care models, psychobehavioral phenotyping may contribute to a broader individualized understanding of therapeutic resistance and patient variability. However, due to the exploratory pre–post design, small sample size, and absence of a control group, causal inferences regarding intervention effectiveness cannot be established. These findings should therefore be interpreted as hypothesis-generating and supportive of future controlled studies incorporating physiological outcomes, ambulatory blood pressure monitoring, longitudinal follow-up, and multidimensional precision medicine approaches.

## Figures and Tables

**Table 1:** Clinical and sociodemographic characteristics of 20 individuals with resistant arterial hypertension. Salvador, Bahia, Brazil, 2023.

Characteristics	Results *n* (%)
Age, years, median (IQR)	55 (49–64)
Female sex	18 (90%)
Afro-descendant skin color	20 (100%)
Secondary Education	15 (75%)
Higher Education	5 (25%)
Without partner	9 (45%)
With partner	11 (55%)
Has children	18 (90%)
Retired/pensioner	9 (45%)
Family income: less than 1 minimum wage	3 (15%)
Family income: 1–2 minimum wages	14 (70%)
Family income: 3–4 minimum wages	3 (15%)
Normal weight	2 (10%)
Overweight	5 (25%)
Obesity	13 (65%)

Note. BMI categorized according to obesity classes defined by the World Health Organization.

## Data Availability

The datasets generated and analyzed during the current study are available from the corresponding author on reasonable request. Due to the sensitive nature of clinical and psychological data, public deposition is restricted to protect participant confidentiality.
